# Impact of COVID-19 control measures on influenza positivity among patients with acute respiratory infections, 2018–2023: an interrupted time series analysis

**DOI:** 10.1186/s12879-025-11279-6

**Published:** 2025-07-18

**Authors:** Wei Chen, Huabin Wang, Xianlin Ten, Miao Fu, Meili Lin, Xiaoping Xu, Yongjun Ma

**Affiliations:** 1 Department of Clinical Laboratory, Affiliated Jinhua Hospital, Zhejiang University School of Medicine, No. 365 Renmin East Road, Jinhua, Zhejiang 321000 China; 2 Department of Clinical Laboratory, Affiliated Jinhua Hospital, Wenzhou Medical University, No. 267 Danxi East Road, Jinhua, Zhejiang 321000 China

**Keywords:** Interrupted time series analysis, Non-pharmaceutical interventions, Influenza epidemiology, Post-pandemic era

## Abstract

**Background:**

After experiencing the global COVID-19 pandemic, whether there have been new changes in the epidemiological characteristics of influenza has become a topic of great concern. This study aims to investigate the impact of implementation and lifting of COVID-19 control measures on influenza positivity among patients with acute respiratory infections (ARI) from 2018 to 2023.

**Methods:**

The data were collected from January 2018 to December 2023 in two designated sentinel hospitals in Jinhua. We performed an interrupted time series analysis (ITSA) using a beta regression model and a generalized additive model (GAM), adopting a two-model cross-validation strategy to assess the effect of two major interventions on influenza positivity: the COVID-19 control measures implemented in early 2020 and lifted at the end of 2022. We also analyzed influenza epidemiological characteristics and seasonality before, during, and after the pandemic.

**Results:**

A total of 98,244 cases were included in this study, and the overall influenza positivity rate was 39.34%. Females and the 6–17-year age group had higher positivity rates. Before the pandemic, influenza primarily showed a winter peak pattern, whereas during the pandemic, the positivity rate declined significantly with no distinct peak. After the pandemic ended, an unusual dual-peak pattern emerged. The interrupted time series analysis revealed that, following the implementation of non-pharmaceutical interventions (NPIs) in early 2020, influenza positivity immediately decreased significantly in the beta regression model (*β* = -1.75, *p* = 0.003). After the lifting of measures in late 2022, a marginally lagged increasing trend was observed in the beta regression model (*β* = 0.14, *p* = 0.096) and a significant increasing trend was found in the GAM model (edf = 7.00, *p* < 0.001). Seasonal effects differed between the models: the beta regression model exhibited significant annual seasonal fluctuations (sin12 = 0.67, *p* < 0.001), while the GAM model did not exhibit a significant association independent of the time trend.

**Conclusion:**

COVID-19 and its control measures substantially reduced influenza positivity rates; however, once these measures were lifted, influenza activity resurged, and its seasonal epidemic pattern changed. The intensity of influenza appeared to exceed pre-pandemic levels, underscoring the importance of NPIs in controlling respiratory infectious diseases. Strengthened surveillance and optimized strategies remain necessary to mitigate the threat of influenza in the post-pandemic era.

**Supplementary Information:**

The online version contains supplementary material available at 10.1186/s12879-025-11279-6.

## Background

Influenza virus belongs to the Orthomyxoviridae family of single-stranded negative-sense RNA viruses and spreads via droplets, aerosols, and contact, to which the general population is widely susceptible [1]. Because the virus easily undergoes antigenic variation, combined with its rapid and widespread transmission, it causes seasonal epidemics almost every year. According to the World Health Organization (WHO), approximately 8% of adults and 25% of children worldwide are infected with influenza annually, leading to nearly 1 billion seasonal influenza cases, among which 3–5 million are severe, resulting in 290,000–650,000 respiratory deaths [2, 3]. China, with a population of 1.4 billion, bears a heavy burden of influenza-associated morbidity and mortality [4], reporting an average of 88,000 influenza deaths annually, accounting for roughly 13.6% of all global influenza deaths [5]. Despite WHO’s continuous efforts to reduce the threat of influenza by increasing vaccine coverage, influenza still poses significant challenges due to seasonal epidemics and periodic outbreaks worldwide.

Toward the end of December 2019 [6], a novel coronavirus was first detected in Wuhan, China, and subsequently spread rapidly to over 200 countries and regions. Because effective vaccines and targeted antivirals were initially lacking, countries worldwide adopted non-pharmaceutical interventions (NPIs) such as lockdowns, travel bans, work-from-home orders, social distancing, mask-wearing, and hand hygiene to curb the spread of COVID-19. These measures were intended to contain the pandemic but also impacted the transmission of other common respiratory pathogens such as influenza and respiratory syncytial virus [7]. By December 2022, as COVID-19 vaccine coverage had risen and viral virulence had weakened, coupled with lower rates of severe disease and mortality, China gradually lifted its NPIs. Nevertheless, three years of the COVID-19 pandemic undoubtedly exerted a profound influence on the epidemiological patterns of respiratory viruses.

Although numerous studies examined changes in influenza epidemiology before and during the pandemic [8–12], whether the lifting of control measures led to a renewed surge or altered epidemic pattern of respiratory pathogens, including influenza, remained a question of concern. Therefore, in this study, we analyzed influenza virus detection data collected from two sentinel hospitals in Jinhua from 2018 to 2023, employing an interrupted time series analysis (ITSA) using a beta regression model and a Generalized Additive Model (GAM) to evaluate the effects of two major interventions implementing COVID-19 control measures in early 2020 and lifting them at the end of 2022—on influenza positivity. We also investigated changes in influenza epidemic patterns, seasonality, and population susceptibility before, during, and after the pandemic, providing insights for targeted influenza control strategies in the post-pandemic era.

## Materials and methods

### Study population

This retrospective study was based on influenza testing in the Jinhua Municipal Central Hospital Medical Group (Hospital A) and Jinhua Municipal People’s Hospital (Hospital B). Hospital A was a tertiary Class-A general hospital with 3,159 open beds, over 2.2 million annual outpatient visits and over 130,000 inpatient admissions, occupying a leading position in Zhejiang’s central regional healthcare network. Hospital B was a tertiary Class-B general hospital with 1,265 open beds, nearly 760,000 annual outpatient visits, and about 70,000 inpatient admissions, playing an important role in healthcare services in central Zhejiang.

The data for this study were retrieved through structured searches in the hospital electronic medical record system (EMR) and laboratory information management system (LIS), with the specific procedures as follows: (1) Professional personnel from the hospital information center extracted influenza nucleic acid testing records of all acute respiratory infection (ARI) patients from two sentinel hospitals between January 1, 2018 and December 31, 2023 using standardized query statements; (2) For patients with multiple test records within 30 days, only the first result was retained; (3) Cases with incomplete data were excluded. The entire data extraction process adhered to the anonymization principle, retaining only non-sensitive fields such as gender, age, and clinical characteristics, while all personally identifiable information was de-identified during data processing.

The ARI was defined as (1) at least one of the following conditions: fever, abnormal white blood cell differentials, leukocytosis or leukopenia; (2) at least one of the following symptoms/signs: cough, chills, expectoration, nasal congestion, sore throat, chest pain, tachypnea, and abnormal pulmonary breath sounds [13]. The diagnosis of pneumonia followed the “Guidelines for the Diagnosis and Treatment of Adult Community-Acquired Pneumonia (2016 version)” by the Chinese Medical Association Respiratory Diseases Branch [14]. Patients were classified into upper respiratory tract infections (URTI) and lower respiratory tract infections (LRTI), with “lower respiratory tract infection”, “bronchitis”, “pneumonia”, and “pulmonary infection” combined under LRTI. This study obtained ethical approval from Affiliated Jinhua Hospital, Zhejiang University School of Medicine, Approval NO: (Research) 2025-Ethical Review-56.

### Specimen collection and laboratory examination

Sterile swabs were used to sample the bilateral palatal arches and pharyngeal and tonsillar areas of the oropharynx, 1–2 times each, with swab heads broken off and placed in virus preservation solution. The tubes were tightly sealed, transported at low temperature, and tested within 24 h.

During the study period from January 2018 to February 2023, the two medical institutions utilized influenza nucleic acid detection reagents from different manufacturers (Hospital A: Shanghai ZJ Bio-Tech Co., Ltd.; Hospital B: Tianlong Tech Co., Ltd.), and both uniformly adopted Shanghai BioGerm Medical Tech Co., Ltd. reagents starting on March 1, 2023. All testing strictly followed the standardized operational procedures specified in the manufacturer’s instructions, employing a three-channel detection system (influenza A/B and internal reference). Result interpretation was based on cycle threshold (CT value) criteria: positive results required internal reference CT values meeting quality control thresholds (ZJ: <36; Tianlong: <40; BioGerm: ≤40), with influenza-specific CT thresholds (ZJ: ≤38; Tianlong: ≤37; BioGerm: ≤39). Borderline samples (ZJ: 38–40; Tianlong: 37–40; BioGerm: 39–45) underwent retesting, and those meeting thresholds upon retest (ZJ: ≤38; Tianlong: <40; BioGerm: <45) were classified as positive. Standardized workflows and uniform quality control protocols ensured consistency. Notably, both hospitals maintained rigorous daily internal quality control protocols and consistently passed annual provincial and national external quality assessments during the study period, which collectively validated the cross-temporal and cross-platform comparability of influenza detection results despite reagent changes.

### Data resource

In this study, we first performed statistical analysis of age data, with the Median (Q1, Q3) showing as: 19(6, 53). Combined with the age group classification from the study by Yan-Ning Liu et al. [15], we classified ages into four groups: ≤5 years (Children), 6–17 years (Adolescents), 18–60 years (Adults), and > 60 years (Older adults). Meanwhile, to investigate the impact of COVID-19 on influenza viruses, the time frames from January 2018 to December 2019 were designated as the first stage (pre-COVID-19 outbreak), January 2020 to December 2022 as the second stage (COVID-19 pandemic period), and January to December 2023 as the third stage (post-COVID-19 pandemic).

### Statistical analysis

In this study, influenza epidemics were described by positivity rates (%) with 95% confidence intervals, using the Wilson score method to derive interval estimates. The relationships between potential variables and influenza positivity rate were expressed as adjusted odds ratios (OR) with 95% confidence intervals, which were calculated using multivariate logistic regression models. Variables adjusted in the logistic regression models (gender, age, and clinical diagnosis) were selected based on prior literature indicating their potential confounding effects on influenza positivity [8]. Additionally, univariate analyses demonstrated significant associations between these variables and the outcome (*p* < 0.05), warranting their inclusion as covariates to control for confounding bias. For instance, when calculating the adjusted OR for “gender”, both “age” and “clinical diagnosis” were included as covariates in the model. Detailed influenza trends were displayed through stacked/line combo plots of monthly positivity rates and heatmaps of weekly positivity rates. Changes in influenza positivity before and after the COVID-19 pandemic were compared using relative risk (RR). For evaluating the impact of the COVID-19 pandemic on influenza viruses, this study employed interrupted time series analysis, enhancing result reliability through a dual-model collaborative analysis: (1) Beta regression model: contains Autoregressive Moving Average (ARMA) components and Fourier seasonal terms, used to quantify the immediate intervention effects and linear lag trends (R package *glmmTMB*); (2) GAM: captures nonlinear temporal interaction effects through smooth functions (R package *mgcv*). Model fitness evaluations was determined according to AIC/BIC criteria and residual autocorrelation tests. SPSS 19.0, GraphPad Prism 9.5.0 and R software (version 4.5.0) were used for all statistical analyses, taking *p* < 0.05 as significant.

## Results

### Basic characteristics and overall influenza positivity rate

From January 2018 to December 2023, 98,244 ARI cases from two designated sentinel hospitals in Jinhua were included. Among them, 38,645 were positive for influenza, resulting in an overall positivity of 39.34% (38,645/98,244; 95% CI: 39.03–39.64). Influenza A virus accounted for 30,299 positive samples (30.84%, 30,299/98,244), whereas Influenza B virus accounted for 8,070 (8.21%, 8,070/98,244) and mixed infections 276 (0.28%, 276/98,244). Regarding gender, females had a higher positivity than males (40.03% vs. 38.70%, *χ²* = 18.18, *p* < 0.001). By age, the 6–17-year group exhibited the highest positivity (54.53%), and the > 60-year group was the lowest (18.64%) (*χ²* = 5907.23, *p* < 0.001). According to clinical classification, URTI cases had a 43.41% positivity, significantly higher than 22.72% in LRTI cases (*χ²* = 2783.81, *p* < 0.001). Detailed data were presented in Table 1.

**Table 1:** The positive rate of influenza virus during 2018 to June 2023

Group	No.(%)	Positive (%)				Positive rate (95% CI), %	Unadjusted OR (95% CI)	*P*	Adjusted OR (95% CI)*	*P*
		Total	Influenza A virus	Influenza B virus	Mixed					
Overall	98,244	38,645(100)	30,299(78.40)	8070(20.88)	276(0.72)	39.34(39.03, 39.64)				
Gender										
Male	51,447(52.37)	19,911(100)	15,530(78.00)	4226(21.22)	155(0.78)	38.70(38.28, 39.12)	Reference		Reference	
Female	46,797(47.63)	18,734(100)	14,769(78.84)	3844(20.52)	121(0.64)	40.03(39.59, 40.48)	1.06(1.03, 1.08)	< 0.001	1.01(0.98, 1.04)^a^	0.534
Age, year										
Children, ≤ 5 years	22,980(23.39)	8509(100)	6441(75.70)	1983(23.30)	85(1.00)	37.03(36.41, 37.65)	Reference		Reference	
Adolescents, 6–17 years	24,442(24.88)	13,329(100)	9869(74.04)	3359(25.20)	101(0.76)	54.53(53.91, 55.16)	2.04(1.97, 2.12)	< 0.001	1.75(1.68, 1.81)^b^	< 0.001
Adults, 18–60 years	31,800(32.37)	13,262(100)	11,054(83.35)	2147(16.19)	61(0.46)	41.70(41.16, 42.25)	1.22(1.17, 1.26)	< 0.001	0.95(0.92, 0.99)^b^	0.006
Older adults, > 60 years	19,022(19.36)	3545(100)	2935(82.79)	581(16.39)	29(0.82)	18.64(18.09, 19.20)	0.39(0.37, 0.41)	< 0.001	0.33(0.31, 0.34)^b^	< 0.001
Diagnosis										
URTI	78,911(80.32)	34,252(100)	27,034(78.93)	6993(20.42)	225(0.65)	43.41(43.06, 43.75)	Reference		Reference	
LRTI	19,333(19.68)	4393(100)	3265(74.32)	1077(24.52)	51(1.16)	22.72(22.14, 23.32)	0.38(0.37, 0.40)	< 0.001	0.37(0.36, 0.38)^c^	< 0.001

*URTI* Upper respiratory tract infection, *LRTI* Lower respiratory tract infection, *CI* Confidence interval, *OR* Odds ratio, *P P* value

*The unadjusted OR results demonstrate that variables (gender, age, clinical diagnosis) are significant associated with influenza virus positivity rates. Consequently, logistic regression should be employed to calculate adjusted OR values to eliminate confounding bias, thereby enabling a more accurate evaluation of the independent effects of each variable

^a^Adjusted for age and diagnosis; ^b^Adjusted for gender and diagnosis

^c^Adjusted for gender and age

Further stratified analysis revealed the associations between various variables and influenza positivity rate. The relationship with gender exhibited a complex pattern: in the 6–17-year age group, females served as a protective factor compared with males, whereas among individuals ≥ 18 years old clinically diagnosed with URTI, females emerged as a risk factor. Regarding age stratification: compared to the ≤ 5 years cohort, the 6–17-year group demonstrated increased risk, while the > 60 years population showed protective effects (except the"LRTI and female” group). In clinical diagnosis classification: LRTI was identified as a protective factor relative to URTI (Supplementary Table 1).

### Influenza positivity trends and seasonal characteristics

**Fig. 1:**
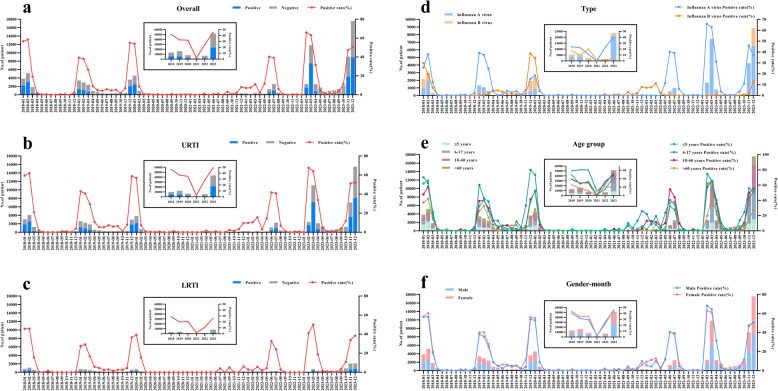
The positive rate of Influenza virus by month during January 2018 to December 2023. URTI, Upper respiratory tract infection, LRTI, Lower respiratory tract infection. **a** The positive rate of Influenza virus among the overall; **b** The positive rate of Influenza virus among patients with URTI; **c** The positive rate of Influenza virus among patients with LRTI; **d** The positive rate of Influenza A virus and Influenza B virus; **e** The positive rate of Influenza virus among the overall patients in diferent age groups; **f** The positive rate of Influenza virus among the overall patients of diferent gender

**Fig. 2:**
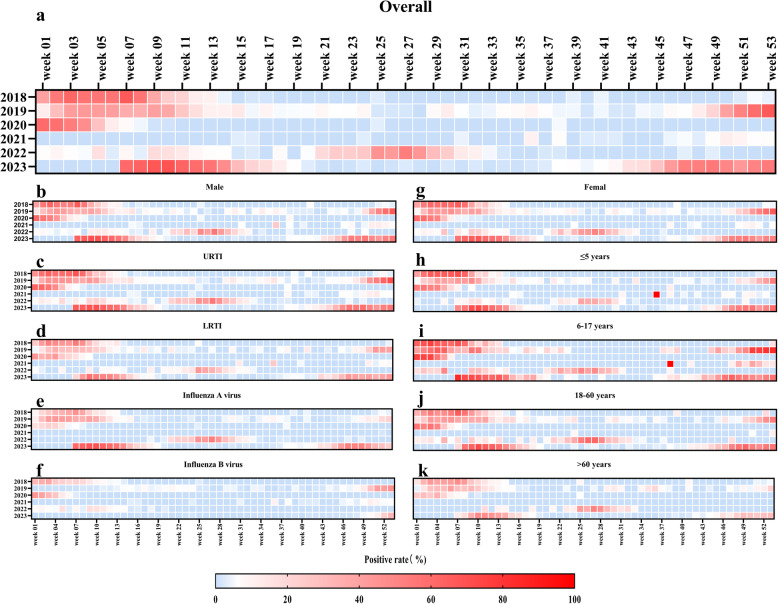
The heatmap of weekly positive rate of Influenza virus among gender, diagnosis, type and age groups from 2018 to 2023. URTI, Upper respiratory tract infection, LRTI, Lower respiratory tract infection. **a** Weekly positive rate of Influenza virus among the overall patients; **b** Weekly positive rate of Influenza virus among the overall male patients; **c** Weekly positive rate of Influenza virus among patients with URTI; **d** Weekly positive rate of Influenza virus among patients with LRTI; **e** Weekly positive rate of Influenza A virus among the overall patients; **f** Weekly positive rate of Influenza B virus among the overall patients; **g** Weekly positive rate of Influenza virus among the overall female patients; **h** Weekly positive rate of Influenza virus among the overall patients aged ≤5 years; **i** Weekly positive rate of Influenza virus among the overall patients aged 6-17years; **j** Weekly positive rate of Influenza virus among the overall patients aged 18-60 years; **k** Weekly positive rate of Influenza virus among the overall patients aged >60 years;

Analyses of annual and monthly influenza positivity from 2018 to 2023 (Fig. 1) indicated that 2018–2019 exhibited winter peaks, whereas 2020–2021 showed a steep decline and absence of a typical peak. In 2022, a summer peak (June–July) reoccurred, while 2023 saw an unusual dual-peak pattern (February-April and November-December). Similar trends were observed in both URTI and LRTI groups. Subtype distribution from 2018 to 2023 revealed that Influenza A virus prevailed overall (78.97%), though Influenza B virus temporarily replaced Influenza A virus as the predominant subtype in 2020–2021. By age group, the 6–17-year group consistently had high positivity, but in 2022, the 18–60-year group briefly surpassed it, becoming the highest-positivity group. Heatmaps of weekly positivity (Fig. 2) underscored substantial variation across years at a finer time scale.

### Comparison of influenza epidemiology before and after the pandemic

Influenza positivity rose from 36.80% in the pre-pandemic phase (2018–2019) to 45.43% in the post-pandemic phase (2023) (RR = 1.23, 95% CI = 1.21–1.26, *p* < 0.001). Among URTI patients, it climbed from 41.01 to 49.12% (RR = 1.20, *p* < 0.001), and among LRTI patients, from 23.91 to 26.14% (RR = 1.09, *p* = 0.001) (Table 2). Analyzing by gender revealed upward trends in both males (35.40–45.98%) and females (38.42–44.85%). By age, ≤ 5 years and 18–60 years increased considerably, whereas 6–17 years and > 60 years decreased, all with statistically significant differences.

**Table 2:** The positive rate of influenza virus before, and after the COVID-19 epidemic

Group	2018–2019			2023				
	No.	Positive	Positive rate (95% CI),%	No.	Positive	Positive rate (95% CI),%	RR (95% CI)	*P*
**Overall**	29,523	10,865	36.80(36.25, 37.35)	51,735	23,504	45.43(45.00, 45.86)	1.23(1.21, 1.26)	< 0.001
Gender								
Male	15,844	5609	35.40(34.66, 36.15)	26,570	12,218	45.98(45.39, 46.58)	1.30(1.27, 1.33)	< 0.001
Female	13,679	5256	38.42(37.61, 39.24)	25,165	11,286	44.85(44.23, 45.46)	1.17(1.14, 1.20)	< 0.001
Age, year								
Children, ≤ 5 years	9425	3670	38.94(37.96, 39.93)	8746	3854	44.07(43.03, 45.11)	1.13(1.09, 1.17)	< 0.001
Adolescents, 6–17 years	4816	2903	60.28(58.89, 61.65)	16,876	9260	54.87(54.12, 55.62)	0.91(0.89, 0.94)	< 0.001
Adults, 18–60 years	8388	2829	33.73(32.72, 34.75)	18,012	8856	49.17(48.44, 49.90)	1.46(1.41, 1.51)	< 0.001
Older adults, > 60 years	6894	1463	21.22(20.27, 22.20)	8101	1534	18.94(18.10, 19.80)	0.89(0.84, 0.95)	< 0.001
**URTI**	22,257	9128	41.01(40.37, 41.66)	43,438	21,335	49.12(48.65, 49.59)	1.20(1.18, 1.22)	< 0.001
Gender								
Male	11,750	4657	39.63(38.75, 40.52)	22,150	11,009	49.70(49.04, 50.36)	1.25(1.22, 1.29)	< 0.001
Female	10,507	4471	42.55(41.61, 43.50)	21,288	10,326	48.51(47.84, 49.18)	1.14(1.11, 1.17)	< 0.001
Age, year								
Children, ≤ 5 years	5950	2889	48.55(47.29, 49.83)	5862	3171	54.09(52.82, 55.37)	1.14(1.08, 1.15)	< 0.001
Adolescents, 6–17 years	4051	2582	63.74(62.24, 65.20)	13,894	8177	58.85(58.03, 59.67)	0.92(0.90, 0.95)	< 0.001
Adults, 18–60 years	7157	2566	35.85(34.75, 36.97)	16,830	8636	51.31(50.56, 52.07)	1.43(13.8, 1.48)	< 0.001
Older adults, > 60 years	5099	1091	21.40(20.29, 22.54)	6852	1351	19.72(18.79, 20.68)	0.92(0.86, 0.99)	0.024
**LRTI**	7266	1737	23.91(22.94, 24.90)	8297	2169	26.14(25.21, 27.10)	1.09(1.04, 1.16)	0.001
Gender								
Male	4094	952	23.25(21.98, 24.57)	4420	1209	27.35(26.04, 28.69)	1.18(1.09, 1.27)	< 0.001
Female	3172	785	24.75(23.28, 26.28)	3877	960	24.76(23.43, 26.14)	1.00(0.92, 1.09)	0.989
Age, year								
Children, ≤ 5 years	3475	781	22.47(21.12, 23.89)	2884	683	23.68(22.17, 25.27)	1.05(0.96, 1.15)	0.255
Adolescents, 6–17 years	765	321	41.96(38.51, 45.49)	2982	1083	36.32(34.61, 38.06)	0.87(0.79, 0.95)	0.004
Adults, 18–60 years	1231	263	21.36(19.17, 23.74)	1182	220	18.61(16.50, 20.93)	0.87(0.74, 1.02)	0.091
Older adults, > 60 years	1795	372	20.72(18.91, 22.66)	1249	183	14.65(12.80, 16.72)	0.71(0.60, 0.83)	< 0.001

*URTI* Upper respiratory tract infection, *LRTI* Lower respiratory tract infection, *CI* Confidence interval, *RR* Relative risk, *P P* value

### Interrupted time series analysis evaluation of the effect of control measures

**Fig. 3:**
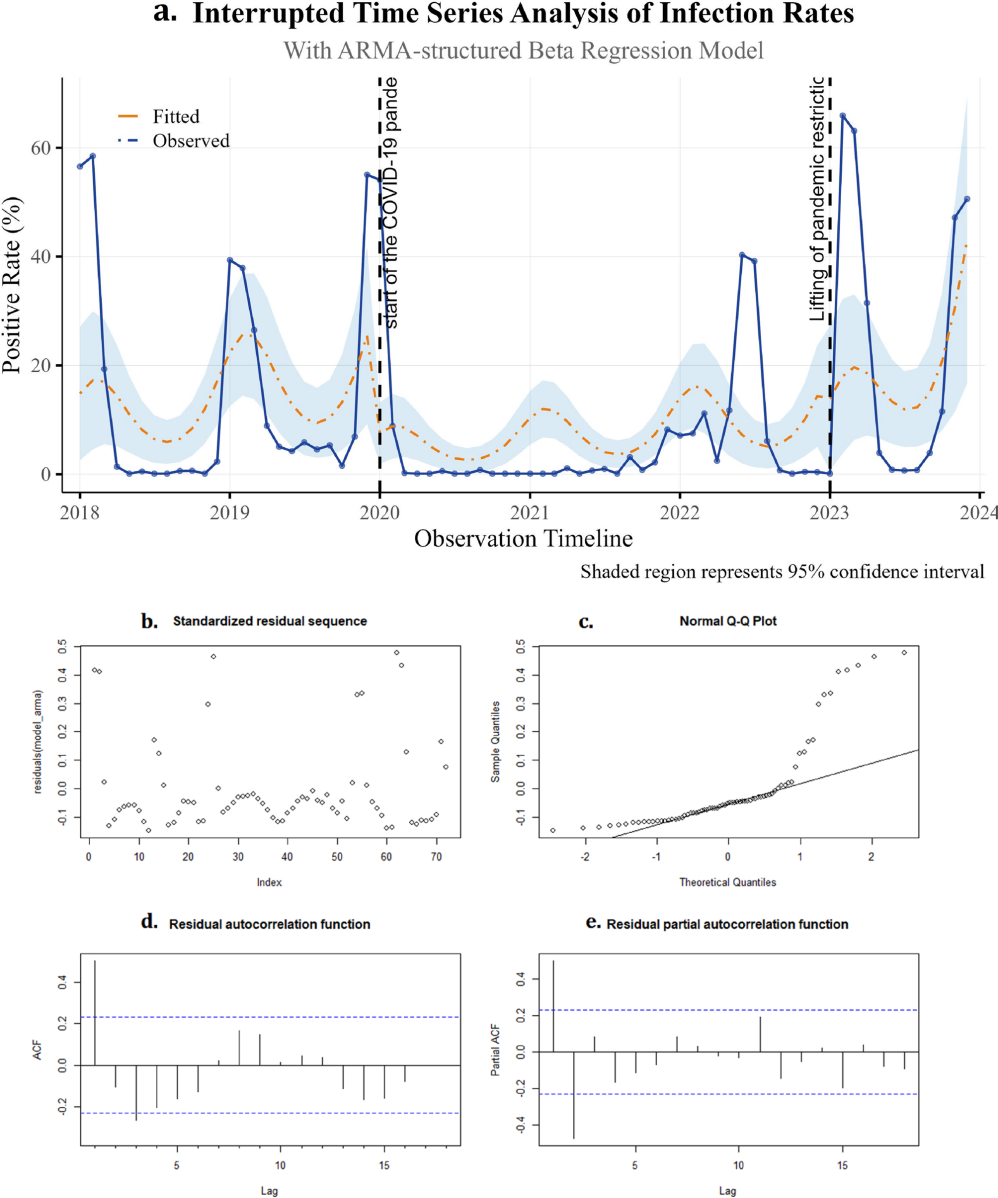
Interrupted time series analysis curves and related model indicators of influenza virus positive rates from 2018 to 2023 base on the beta regression model. **a** Interrupted time series analysis curve. **b** Standardizedresidual sequence **c** Normal Q-QPlot **d** Residual autocorrelation function. **e** Residual partial autocorrelation function. The blue solid line represents the actual observed values, while the orange dashed line represents the model-predicted values. The vertical dashed line marks the implementation and removal time points of epidemic prevention and control measures

To systematically assess the dynamic impact of NPIs on influenza positivity rates, this study employed complementary analyses using a beta regression model and a GAM model. The beta regression results (Table 3) showed that Intervention 1 (implementation of NPIs in early 2020) resulted in a significant immediate decrease in influenza positivity (*β* = −1.75, *p* = 0.003). Additionally, the lifting of NPIs at the end of 2022 resulted in a significant immediate effect (*β* = −2.24, *p* = 0.029), though its lag term suggested a marginal upward trend post-intervention (*β* = 0.14, *p* = 0.096). The model also identified an annual cycle with winter dominance (sin12 *β* = 0.67, *p* < 0.001; cos12 *β* = 0.30, *p* = 0.090). Despite satisfactory overall model fit (AIC = −246.9, BIC= −224.2), residual autocorrelation at lag 6 (*p* < 0.001) and lag 12 (*p* < 0.001) indicated incomplete control of temporal dependence. Interrupted time series analysis curves and related model indicators of influenza virus positive rates from 2018 to 2023 (Fig. 3) displayed the observed data versus model forecasts over time.

**Table 3:** Beta regression model results of influenza positive rate in 2018–2023 affected by time, COVID-19 epidemic and seasonality

Variable	Estimate (β)	Std. Error	z value	*p* value	95% CI
Intercept	−2.38	0.49	−4.87	< 0.001	(−3.34, −1.42)
Time	0.04	0.03	1.28	0.200	(−0.02, 0.11)
Intervention 1	−1.75	0.59	−2.94	0.003	(−2.92, −0.58)
Time After Intervention 1	−0.01	0.04	−0.37	0.711	(−0.08, 0.06)
Intervention 2	−2.24	1.03	−2.18	0.029	(−4.27, −0.21)
Time After Intervention 2	0.14	0.08	1.66	0.096	(−0.03, 0.30)
sin12 (Seasonal Term)	0.67	0.20	3.41	< 0.001	(0.28, 1.05)
cos12 (Seasonal Term)	0.30	0.17	1.70	0.090	(−0.05, 0.64)
Phi (Precision Parameter)	1.40	0.34	4.10	< 0.001	(0.73, 2.08)

Intervention 1: The implementation of COVID-19 prevention and control measures in early 2020

Intervention 2: The lifting of COVID-19 prevention and control measures at the end of 2022

The GAM model (Supplementary Table 2) further revealed a significant nonlinear increasing trend in positivity after the lifting of NPIs (s(Time_After_Intervention_2): edf = 7.00, *p* < 0.001). Additionally, testing volume (log_tests) exhibited confounding effects on intervention outcomes (baseline edf = 7.04 vs. intervention-period edf = 6.21, *p* < 0.001), suggesting potential distortion in policy effect estimation. Although autocorrelation correction (*ρ* = 0.3) in the GAM model partially mitigated temporal dependence, the high degrees of freedom in spline terms (total edf = 68) raised overfitting concerns (adjusted R² = 0.985).

The cross-model analysis confirmed that NPIs implementation generated immediate suppression, while post-cessation positivity rebounded through nonlinear trends (RR = 1.23, *p* < 0.001). The beta regression’s independent seasonal signal (winter peak) complemented the GAM model’s time-trend-absorbed seasonal pattern (s(Months): *p* = 0.009), highlighting methodological influences on result interpretation. Residual diagnostics from both models underscored the need for cautious interpretation of temporal dependencie.

A further comparison of weekly positivity across pre-pandemic (Stage I), pandemic (Stage II), and post-pandemic (Stage III) phases (Supplementary Fig. 1) revealed that positivity during Stage II was distinctly lower than in Stage I or Stage III, and Influenza A virus during the post-pandemic stage was notably higher than pre-pandemic.

## Discussion

This study examined influenza detection results from 2018 to 2023 among ARI patients who visited two designated sentinel hospitals in Jinhua. The overall positivity among these ARI patients was 39.34%, which was close to the global median influenza positivity of 38.40% from 2014 to 2019 [16], lower than the 49.60% (± 8.5) reported in Belgium from 2009 to 2019 [17], but higher than the 20.64% found in Pakistan from 2008 to 2017 [18]. Marked annual disparities were evident, with a year-by-year decrease from 2018 to 2020 (41.86%, 32.64%, 32.13%) until it dropped to a historical low of 2.03% in 2021 amid the COVID-19 pandemic. When control measures in China transitioned to a normalized phase in 2022, the influenza positivity recovered to 24.62%, and once the pandemic ended in 2023, it reached a peak of 45.53%. This result is consistent with the annual variations in global influenza positivity rates reported by the Global Influenza Surveillance and Response System (GISRS) [16].

By gender, in alignment with earlier research [19], female positivity (40.03%) significantly exceeded male positivity (38.70%). Although the underlying reasons remained uncertain, immunologic differences were believed to exist between the sexes, and women typically exhibited stronger immune responses to infections and vaccines [20, 21]. Concerning age distribution, findings from previous sources indicated that the highest influenza positivity appeared in the 5–14-year group (41.70%), and the lowest in the 30–60-year and ≥ 65-year adult groups (30.70%) [22]. In this study, 6–17-year group (54.53%) was the highest, and > 60 year group (18.64%) was the lowest. Possible explanations included incomplete immune system development among adolescents and more active social contact, as well as incomplete adherence to hygiene protocols.

The influenza virus is generally regarded as a seasonal epidemic respiratory virus, yet it manifested varying patterns by latitude and geography. In most mid-to-low latitude regions of China, influenza was known to circulate semiannually (e.g., January–February; June–August in Zhejiang and Anhui) or even year-round, such as in Hainan, China [23, 24]. Although Jinhua lay in a mid-subtropical area of Zhejiang Province, the findings revealed altered seasonal patterns at different stages of pandemic intervention.

Before the first intervention (implementation of pandemic control measures), the epidemic pattern was consistent with the influenza epidemic characteristics in Zhejiang Province during the same period as reported by Wu Haocheng et al. [3], both showing a single-peak pattern in winter. However, once stringent NPIs were enforced, the observed values fell substantially below the interrupted time series analysis model projections, and winter peaks vanished or were severely suppressed in 2020–2021. This phenomenon was consistent with reports from numerous countries [9, 10, 12, 25, 26], suggesting that NPIs not only controlled COVID-19 but also dramatically curtailed influenza activity. As data on costs and benefits of NPIs accumulated, it was considered possible to adopt context-specific interventions to manage epidemic diseases like influenza [27, 28]. For instance, during epidemic seasons, mild “mask policies” are implemented in crowded enclosed public places such as hospitals and railway stations; semi-closed management measures are adopted in institutions primarily serving vulnerable populations such as childcare centers and nursing homes, to reduce the burdens caused by these viral infections.

Upon lifting control measures at the end of 2022, which constituted the second intervention, the previously suppressed influenza positivity rebounded sharply, producing two peaks in 2023. Interestingly, these peaks were not the usual “winter-plus-summer” double peaks characteristic of mid-latitude climates, but instead featured two winter peaks, including a delayed 2022 winter peak and an earlier 2023 winter peak. This observation suggested that while the pandemic response had only postponed influenza transmission, the virus resurged vigorously once those measures ended [7]. It also implied that seasonal influenza patterns had been disrupted and reestablished post-pandemic [16]. Will the emergent epidemic pattern persist? Alternatively, will it gradually return to pre-pandemic norms after several epidemic seasons, similar to the respiratory syncytial virus (RSV) following the 2009 influenza pandemic [29]? Further epidemiological evidence is needed to confirm this trend. The change in epidemic patterns presents new challenges for influenza surveillance. Compared with the “targeted surveillance” model during traditional epidemic seasons, strengthening “year-round surveillance” may be a more suitable strategy.

With the complementary design of the beta regression model and the GAM model, this study aims to balance the interpretability of the parametric model for immediate effects with the dynamic trend capturing ability of the nonparametric model. Parametric estimates in beta models (e.g., Intervention 1: *β*= −1.75, *P* = 0.003) provide explicit quantitative evidence of the immediate effects of policies, which meets the need for direct interpretation of effect values in public health policymaking, whereas nonlinear dynamics in GAM models [e.g., s(Time_After_Intervention_2): Intervention phase, edf = 7.00, *P* < 0.001] reveal a delayed cumulative effect of the policy, consistent with the lagged response mechanism of interventions in the theory of the dynamics of infectious disease transmission; and inter-model differences regarding seasonal results highlight the influence of methodological choices on the interpretation of results. This overlap and divergence of multi-model results suggests the need to incorporate domain knowledge for model selection in policy evaluation [30]: the intuitive parameters of beta regression models are advantageous when focusing on short-term immediate effects [31, 32]; while the flexibility of GAM models may be more appropriate when exploring long-term complex dynamic trends [33]. This study provides a paradigm for multi-model synergistic analyses, and subsequent studies may incorporate propagation dynamics parameters (e.g., the fundamental regeneration number R0) as confounders to increase biological consistency among models.

Additionally, compared with the pre-pandemic stage (Stage I), infection risk of influenza increased by 23% in the post-pandemic stage (Stage III), with a 30% rise in males and a 46% rise in the 18–60-year age group. The primary drivers might included: (1) Immune deficits due to an absence of natural influenza exposure under NPIs, leading to “immunity debt” [34, 35]. (2) Different peak outbreaks triggered by distinct viral subtypes/strains, such as H3N2 dominating in summer peaks and various pathogens in winter [3]. The subtype/strain cycling further elevated influenza epidemic risks due to antigenic differences. (3) During COVID-19, the government greatly expanded nucleic acid testing capacities and set standardized workflows for SARS-CoV-2, which remained after the pandemic ended, making influenza nucleic acid testing more accessible [36]. The fragile herd immunity, variable subtype/strain circulation, and strong nucleic acid testing capacity jointly promoted the active prevalence of influenza after the pandemic.

Apart from significant threats to health, influenza also imposes substantial economic burdens at both individual and national levels [37, 38]. Influenza vaccination was considered a cost-effective approach to reduce influenza-related morbidity and mortality [39]. However, China’s National Immunization Program did not incorporate influenza vaccines, and only a few affluent regions (Beijing, Shanghai, and Zhejiang Province) provided free vaccination for older adults over 60 years [24]. A 2014–2015 survey reported that China’s overall influenza vaccination coverage stood at 2.4%, with only 3.8% for those > 60 years [40], which was much lower than the 48.4% among U.S. adults [4], let al.one the WHO recommendation of 75.0%. As the post-COVID-19 period saw a strong influenza resurgence, government authorities should consider integrating children, older adults, and healthcare personnel into the national influenza immunization program and promoting combination vaccination to enhance service efficiency [41], thereby raising population-level coverage. Additionally, initiating vaccination within 1–2 months prior to the onset of the epidemic season is often considered the optimal strategy [42]; however, with the new changes in influenza epidemic patterns, determining the appropriate timing for vaccine administration will become more complex. Therefore, six-monthly vaccination may represent an appropriate alternative strategy in vulnerable populations [43].

There were several limitations in this study. First, the study’s data were collected solely from two sentinel hospitals in Jinhua City, Zhejiang Province, which may limit the generalizability of the findings to other regions. Second, the dataset included only ARI patients who actively sought medical care and underwent nucleic acid testing for influenza viruses, introducing potential selection bias. During the COVID-19 pandemic, patients with mild symptoms often opted for home quarantine instead of seeking medical treatment; Additionally, prior to the pandemic, some ARI patients preferred antigenic methods over RT-PCR for influenza testing; These omissions possibly underestimate the positivity rates of influenza among API patients. Third, despite employing RT-PCR—a method recognized for its high sensitivity and specificity—it is not infallible; factors such as specimen quality, target sequence variation, low viral load, and human factors may lead to false negatives or positives. Furthermore, the use of different influenza diagnostic kits across hospitals and periods, with varying threshold interpretations, may have influenced the results. Finally, before the pandemic, PCR-based influenza testing was rarely used; during the pandemic, however, the marked improvement in hospitals’ nucleic acid testing capacity drove the widespread adoption of PCR for influenza detection, resulting in a substantial expansion of the detection base. This temporal disparity in testing scale may introduce bias. Future research could adopt multi-center, larger-scale data collection and consider more factors, such as vaccination rates, population immunity status, and environmental influences, to facilitate more comprehensive influenza control strategies in the post-pandemic era.

## Conclusion

An interrupted time series analysis was employed in this study to evaluate the impact of COVID-19 control measures implemented in early 2020 and lifted at the end of 2022 on influenza transmission. The findings demonstrated that these measures effectively lowered influenza positivity and kept it at a low level for a certain period. Nevertheless, once the measures were lifted, influenza positivity rose again and exhibited a seasonal pattern differing from that before the pandemic. This phenomenon underscored that non-pharmaceutical interventions were effective not only for containing COVID-19 but also for indirectly controlling other respiratory pathogens like influenza. The resurgence of influenza and its altered circulation after the pandemic underscored the need to strengthen influenza monitoring, optimize vaccination strategies, maintain basic preventive measures, and apply NPIs flexibly, thereby minimizing the potential negative impacts on society, the economy, and public health.

## Supplementary Information

Supplementary Material 1.

## Data Availability

The datasets used and analyzed during the current study is available from the corresponding author Wei Chen(E-mail: knight19861120@sina.com) on reasonable request.

## Abbreviations

ARI: Acute respiratory infections
ITSA: Interrupted time series analysis
GAM: Generalized Additive Model
NPIs: Non-pharmaceutical interventions
URTI: Upper respiratory tract infections
LRTI: Lower respiratory tract infections
OR: Odds ratios
RR: Relative risk
95% CI: 95% confidence interval
