# Supplementary material for: Impact of COVID-19 control measures on influenza positivity among patients with acute respiratory infections, 2018–2023: an interrupted time series analysis
Source: BMC Infect Dis. 2025 Jul 18;25:925. doi: 10.1186/s12879-025-11279-6 (PMC12275363; doi:10.1186/s12879-025-11279-6)
Supplement: Supplementary file 1 — Supplementary Material 1. [file 12879_2025_11279_MOESM1_ESM.docx]

**Supplemental data**

**Supplementary Table 1** Stratified analysis of the association between gender, age, clinical diagnosis, and influenza positivity rate

| Group | Variable | Total | Positive | Positive rate (95% CI), % | OR (95% CI) | *P* |
| --- | --- | --- | --- | --- | --- | --- |
| URTI and Children, ≤5 years | Male | 8080 | 3723 | 46.08(44.99, 47.17) | Reference |  |
|  | Female | 6558 | 3122 | 47.61(46.40, 48.82) | 1.06(1.00, 1.14) | 0.065 |
|  |  |  |  |  |  |  |
| URTI and Adolescents, 6-17 years | Male | 11126 | 6640 | 59.68(58.77, 60.59) | Reference |  |
|  | Female | 9070 | 5170 | 57.00(55.98, 58.02) | 0.90(0.85, 0.95) | <0.001 |
|  |  |  |  |  |  |  |
| URTI and Adults, 18-60 years | Male | 12674 | 5485 | 43.28(42.42, 44.14) | Reference |  |
|  | Female | 16173 | 7206 | 44.56(43.79, 45.32) | 1.05(1.01, 1.10) | 0.030 |
|  |  |  |  |  |  |  |
| URTI and Older adults, >60 years | Male | 8942 | 1615 | 18.06(17.28, 18.87) | Reference |  |
|  | Female | 6288 | 1291 | 20.53(19.55, 21.55) | 1.17(1.08, 1.27) | <0.001 |
|  |  |  |  |  |  |  |
| LRTI and Children, ≤5 years | Male | 4634 | 948 | 20.46(19.32, 21.64) | Reference |  |
|  | Female | 3708 | 716 | 19.31(18.07, 20.61) | 0.93(0.83, 1.04) | 0.192 |
|  |  |  |  |  |  |  |
| LRTI and Adolescents, 6-17 years | Male | 2250 | 859 | 38.18(36.19, 40.20) | Reference |  |
|  | Female | 1996 | 660 | 33.07(31.04, 35.16) | 0.80(0.71, 0.91) | 0.001 |
|  |  |  |  |  |  |  |
| LRTI and Adults, 18-60 years | Male | 1393 | 267 | 19.17(17.19, 21.32) | Reference |  |
|  | Female | 1560 | 304 | 19.49(17.60, 21.53) | 1.02(0.85, 1.23) | 0.826 |
|  |  |  |  |  |  |  |
| LRTI and Older adults, >60 years | Male | 2348 | 374 | 15.93(14.50, 17.46) | Reference |  |
|  | Female | 1444 | 265 | 18.35(16.44, 20.43) | 1.19(1.00, 1.41) | 0.053 |
|  |  |  |  |  |  |  |
| URTI and Male | Children, ≤5 years | 8080 | 3723 | 46.08(44.99, 47.17) | Reference |  |
|  | Adolescents, 6-17 years | 11126 | 6640 | 59.68(58.77, 60.59) | 1.73(1.63, 1.84) | <0.001 |
|  | Adults, 18-60 years | 12674 | 5485 | 43.28(42.42, 44.14) | 0.89(0.84, 0.94) | <0.001 |
|  | Older adults, >60 years | 8942 | 1615 | 18.06(17.28, 18.87) | 0.26(0.24, 0.28) | <0.001 |
|  |  |  |  |  |  |  |
| URTI and Female | Children, ≤5 years | 6558 | 3122 | 47.61(46.40, 48.82) | Reference |  |
|  | Adolescents, 6-17 years | 9070 | 5170 | 57.00(55.98, 58.02) | 1.46(1.37, 1.56) | <0.001 |
|  | Adults, 18-60 years | 16173 | 7206 | 44.56(43.79, 45.32) | 0.88(0.83, 0.94) | <0.001 |
|  | Older adults, >60 years | 6288 | 1291 | 20.53(19.55, 21.55) | 0.28(0.26, 0.31) | <0.001 |
|  |  |  |  |  |  |  |
| LRTI and Male | Children, ≤5 years | 4634 | 948 | 20.46(19.32, 21.64) | Reference |  |
|  | Adolescents, 6-17 years | 2250 | 859 | 38.18(36.19, 40.20) | 2.40(2.15, 2.68) | <0.001 |
|  | Adults, 18-60 years | 1393 | 267 | 19.17(17.19, 21.32) | 0.92(0.79, 1.07) | 0.293 |
|  | Older adults, >60 years | 2348 | 374 | 15.93(14.50, 17.46) | 0.74(0.65, 0.84) | <0.001 |
|  |  |  |  |  |  |  |
| LRTI and Female | Children, ≤5 years | 3708 | 716 | 19.31(18.07, 20.61) | Reference |  |
|  | Adolescents, 6-17 years | 1996 | 660 | 33.07(31.04, 35.16) | 2.06(1.82, 2.34) | <0.001 |
|  | Adults, 18-60 years | 1560 | 304 | 19.49(17.60, 21.53) | 1.01(0.87, 1.17) | 0.882 |
|  | Older adults, >60 years | 1444 | 265 | 18.35(16.44, 20.43) | 0.94(0.80, 1.10) | 0.432 |
|  |  |  |  |  |  |  |
| Male and Children, ≤5 years | URTI | 8080 | 3723 | 46.08(44.99, 47.17) | Reference |  |
|  | LRTI | 4634 | 948 | 20.46(19.32, 21.64) | 0.30(0.28, 0.33) | <0.001 |
|  |  |  |  |  |  |  |
| Male and Adolescents, 6-17 years | URTI | 11126 | 6640 | 59.68(58.77, 60.59) | Reference |  |
|  | LRTI | 2250 | 859 | 38.18(36.19, 40.20) | 0.42(0.38, 0.46) | <0.001 |
|  |  |  |  |  |  |  |
| Male and Adults, 18-60 years | URTI | 12674 | 5485 | 43.28(42.42, 44.14) | Reference |  |
|  | LRTI | 1393 | 267 | 19.17(17.19, 21.32) | 0.31(0.27, 0.36) | <0.001 |
|  |  |  |  |  |  |  |
| Male and Older adults, >60 years | URTI | 8942 | 1615 | 18.06(17.28, 18.87) | Reference |  |
|  | LRTI | 2348 | 374 | 15.93(14.50, 17.46) | 0.86(0.76, 0.97) | 0.016 |
|  |  |  |  |  |  |  |
| Female and Children, ≤5 years | URTI | 6558 | 3122 | 47.61(46.40, 48.82) | Reference |  |
|  | LRTI | 3708 | 716 | 19.31(18.07, 20.61) | 0.26(0.24, 0.29) | <0.001 |
|  |  |  |  |  |  |  |
| Female and Adolescents, 6-17 years | URTI | 9070 | 5170 | 57.00(55.98, 58.02) | Reference |  |
|  | LRTI | 1996 | 660 | 33.07(31.04, 35.16) | 0.37(0.34, 0.41) | <0.001 |
|  |  |  |  |  |  |  |
| Female and Adults, 18-60 years | URTI | 16173 | 7206 | 44.56(43.79, 45.32) | Reference |  |
|  | LRTI | 1560 | 304 | 19.49(17.60, 21.53) | 0.30(0.26, 0.34) | <0.001 |
|  |  |  |  |  |  |  |
| Female and Older adults, >60 years | URTI | 6288 | 1291 | 20.53(19.55, 21.55) | Reference |  |
|  | LRTI | 1444 | 265 | 18.35(16.44, 20.43) | 0.87(0.75, 1.01) | 0.063 |

**URTI**, Upper respiratory tract infection, **LRTI**, Lower respiratory tract infection, **CI**, Confidence interval, **OR**, Odds ratio, ***P*** *P* value

**Supplementary Table 2**. Generalized additive model (GAM) results of influenza positive rate in 2018-2023 affected by time, COVID-19 epidemic, seasonality and number of tests

| Variable | Estimate (*β*) | Std. Error | edf | Ref.df | *t* value/*F* | *p* value |
| --- | --- | --- | --- | --- | --- | --- |
| Fixed effects |  |  |  |  |  |  |
| Intercept | 14.65 | 25.19 |  |  | 0.58 | 0.564 |
| Intervention 1 | -4.11 | 23.39 |  |  | -0.18 | 0.861 |
| Intervention 2 | -65.59 | 56.57 |  |  | -1.16 | 0.253 |
| sin12 (Seasonal Term) | 0.84 | 0.70 |  |  | 1.20 | 0.237 |
| cos12 (Seasonal Term) | 0.01 | 0.71 |  |  | 0.01 | 0.991 |
| Smooth terms |  |  |  |  |  |  |
| s(Months) |  |  | 5.98 | 6.90 | 3.21 | 0.009 |
| s(Time_After_Intervention_1): Baseline phase |  |  | <0.01 | <0.01 | 0.09 | 1.000 |
| s(Time_After_Intervention_1): Intervention phase |  |  | 1.00 | 1.00 | 0.05 | 0.822 |
| s(Time_After_Intervention_2): Baseline phase |  |  | <0.01 | <0.01 | 0.000 | 1.000 |
| s(Time_After_Intervention_2): Intervention phase |  |  | 7.00 | 7.32 | 16.48 | <0.001 |
| s(log_tests): Intervention_1 Baseline phase |  |  | 7.04 | 7.81 | 99.20 | <0.001 |
| s(log_tests): Intervention_1 ntervention phase |  |  | 6.21 | 6.78 | 66.24 | <0.001 |

**Intervention 1**: The implementation of COVID-19 prevention and control measures in early 2020

**Intervention 2**: The lifting of COVID-19 prevention and control measures at the end of 2022

**S():** Smooth functions

**Edf**: Effective degrees of freedom (reflects non-linearity when >1)

**Supplementary Figure 1** The weekly positive rate of Influenza virus before, during and after COVID-19 from 2018 to 2023.

**
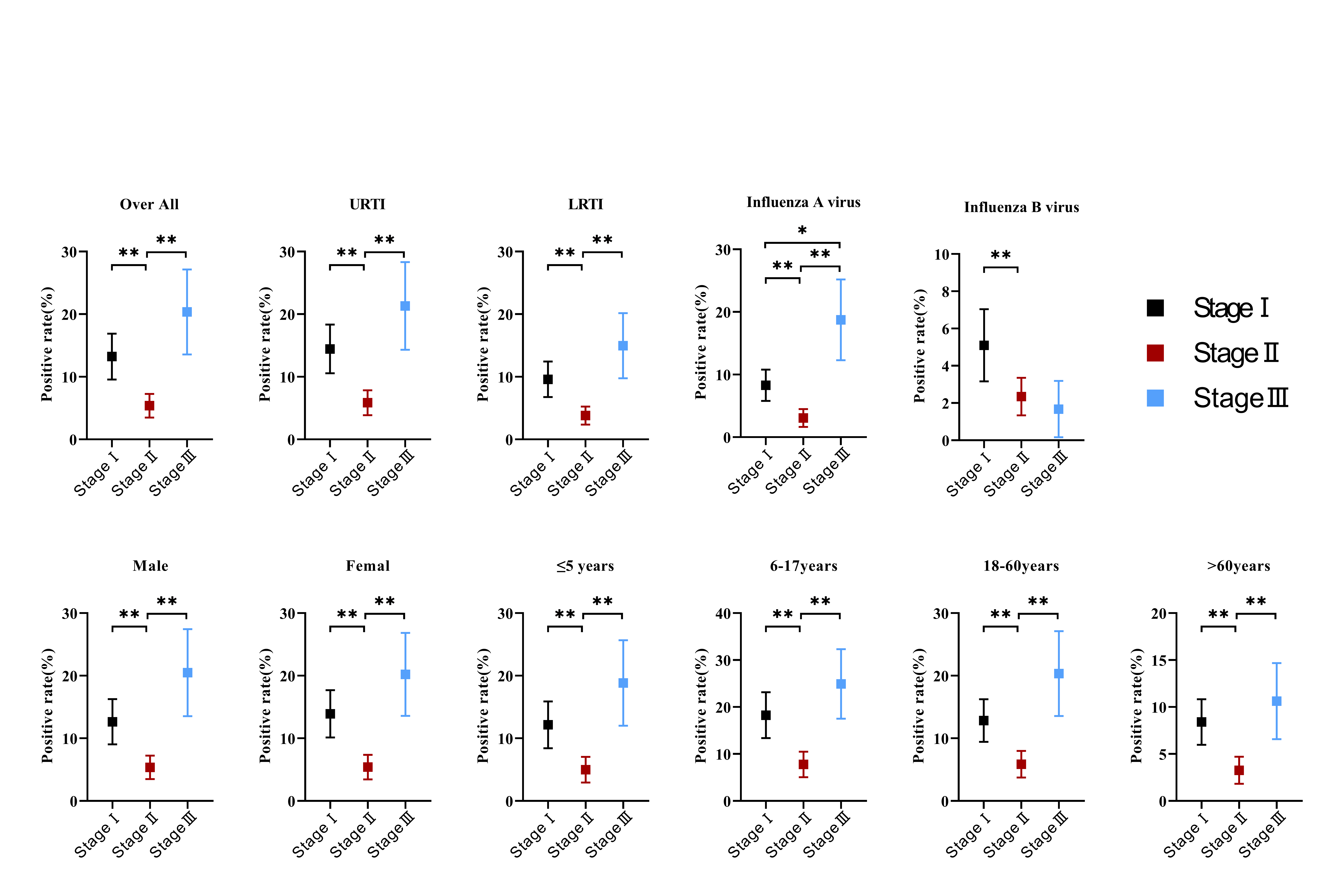
**

**URTI**, Upper respiratory tract infection, **LRTI**, Lower respiratory tract infection

**StageⅠ**: Before the outbreak of the COVID-19, January-December of 2018 to 2019;

**StageⅡ**: During the outbreak of the COVID-19, January-December of 2020 to 2022;

**StageⅢ**: After the outbreak of the COVID-19, January-December of 2023.

"*****" was represented *p*<0.05; "******"was represented *p*<0.001.
